# The Management of the Placenta

**Published:** 1871

**Authors:** 


### Article III.—*The Management of the Placenta.*

Dr. Chantreuil has inserted an essay in the "*Archiv. de Med.*" (October, November, and December, 1870), which he entitles, "Uterine Expression." We are all acquainted with Dr. Hicks's plan of combined external and internal manipulations in certain stages of parturition; here, however, we are advised to compress the fundus of the uterus steadily after the expulsion of the fœtus, so as in some degree to express the placenta, as a cherry-stone is expressed from the fruit. The essay of the author is extremely well written, and his quotations prove his reading to be very extensive. He points out the dangers of pulling upon the cord, and the risk the mother runs when the hand is introduced into the uterine cavity. But he does not allow sufficient importance to the spontaneous extrusion. Dr. Chantreuil reminds his readers that R. Wallace Johnson, in 1769, recommended a pressure of the same kind, combined with some traction of the cord. But Prof. Crede, of Leipsig, is the first who advised the practice in all cases. Dr. Chantreuil has collected no less than 540 cases in which uterine expression has been used by himself, and found that the placenta came away thirty-two times immediately after parturition; seventy-eight times one minute afterwards; 175 times in two minutes; 109 times in three minutes; fifty times in four minutes; forty-seven times in five minutes; twenty times in seven minutes; four times in eight minutes; eleven times in ten minutes; three times in twenty minutes; and once in twenty-five minutes.—*Richmond and Louisville Medical Journal.*

